# Characterization and functional properties of gelatin from goat bone through alcalase and neutrase enzymatic extraction

**DOI:** 10.14202/vetworld.2021.2397-2409

**Published:** 2021-09-17

**Authors:** Dellen Naomi Matulessy, Yuny Erwanto, Nurliyani Nurliyani, Edi Suryanto, Mohammad Zainal Abidin, Thoyib Rohman Hakim

**Affiliations:** Department of Animal Products Technology, Faculty of Animal Sciences, Universitas Gadjah Mada, Jl. Fauna No. 3, Bulaksumur, Yogyakarta 55281, Indonesia.

**Keywords:** alcalase, enzymatic extraction, gelatin quality, Indonesia *Kacang* goat bone, neutrase

## Abstract

**Background and Aim:**

Gelatin is a dissolved protein that results from partial extraction of collagen, commonly from pig and bovine skin. There was no study on gelatin production from *Kacang* goat bones through enzymatic extraction. This study aimed to evaluate the chemical, physical, and functional properties of gelatin from bones of *Kacang* goat using alcalase and neutrase enzymes.

**Materials and Methods:**

Male *Kacang* goat bones aged 6-12 months and two commercial enzymes (alcalase and neutrase) were used for this study. Descriptive analysis and completely randomized design (one-way analysis of variance) were used to analyze the chemical, physical, and functional properties of gelatin. *Kacang* goat bone was extracted with four concentrations of alcalase and neutrase enzymes, namely, 0 U/g (AG-0 and NG-0), 0.02 U/g (AG-1 and NG-1), 0.04 U/g (AG-2 and NG-2), and 0.06 U/g (AG-3 and NG-3) with five replications.

**Results:**

The highest yield of gelatin extraction with alcalase obtained on AG-3 was 9.78%, and that with neutrase on NG-3 was 6.35%. The moisture content of alcalase gelatin was 9.39-9.94%, and that of neutrase gelatin was 9.15-9.24%. The ash and fat content of gelatin with alcalase was lower than that without enzyme treatment with higher protein content. The lowest fat content was noted in AG-1 (0.50%), with protein that was not different for all enzyme concentrations (69.65-70.21%). Gelatin with neutrase had lower ash content than that without neutrase (1.61-1.90%), with the highest protein content in NG-3 (70.89%). The pH of gelatin with alcalase and neutrase was 6.19-6.92 lower than that without enzymes. Melting points, gel strength, and water holding capacity (WHC) of gelatin with the highest alcalase levels on AG-1 and AG-2 ranged from 28.33 to 28.47°C, 67.41 to 68.14 g bloom, and 324.00 to 334.67%, respectively, with viscosity that did not differ, while the highest foam expansion (FE) and foam stability (FS) were noted in AG-1, which were 71.67% and 52.67%, respectively. The highest oil holding capacity (OHC) was found in AG-2 (283%). FS and OHC of gelatins with the highest neutrase levels in NG-2 were 30.00% and 265.33%, respectively, while gel strength, viscosity, FE, and WHC of gelatins with the highest neutrase levels did not differ with those without enzymes at all enzyme concentrations. B chain was degraded in all gelatins, and high-intensity a-chains in gelatin with alcalase and peptide fraction were formed in gelatin with neutrase. Extraction with enzymes showed loss of the triple helix as demonstrated by Fourier transform infrared spectroscopy.

**Conclusion:**

Based on the obtained results, the *Kacang* goat bone was the potential raw source for gelatin production. Enzymatic extraction can increase the quality of gelatin, especially the alcalase (0.02-0.04 U/g bone) method. This can be used to achieve the preferable quality of gelatin with a higher yield.

## Introduction

Gelatin is a product obtained from the hydrolysis of collagen, which is rich in amino acids and has physicochemical properties with functional characteristics and establishes diverse benefits, such as edible film [1], antioxidants [2], stabilizers, emulsifiers for ice cream and yogurt production [3,4], and bioactive functions [5-7]. It can be hydrolyzed from animal textures or by-products that accommodate collagen substances, such as bones, tendons, and skin [8-10]. Hydrolysis of gelatin by exploring the source of bone raw material, with various extraction methods, has been conducted, mostly from fish and some from poultry and mammals [11-15]. *Kacang* goats are native Indonesian goats, widely spread in many regions of Indonesia, and usually kept traditionally for meat. Male goats are preferred by consumers [16]. The bones from *Kacang* goat can be used alternatively as a potential source of gelatin production. The use of bone as a raw material for gelatin production, especially from mammalian bone, was rare, in consequence of the solidity and hardness of the bone structure, which involves long treatment. According to Ma *et al*. [17], the traditional bone gelatin extraction method takes 20-60 days. In contrast, a bone also has a high ash content so that, if it is not pretreated with appropriate methods, it would significantly affect most physical, chemical, and functional characteristics of gelatin. It is interesting to obtain gelatin with the right extraction method according to bone raw material and one of the potential methods in gelatin extraction through the enzymatic curing process.

Enzymatic extraction in gelatin production is widely used. Enzymes are specific and effective in small quantities under milder reaction conditions [18], and gelatin with peptides that have high molecular weight (MW) is obtained to support its functionality [19]. Natural or commercial protease enzymes derived from plants, animals, and microorganisms have been shown to break down the collagen cross-linking by various extraction methods and gelatin production with various characteristics that are directly responsible for the physical, chemical, and functional properties of gelatin [20]. Enzymes with certain conditions can be used as a pretreatment of raw materials before extraction [17,21,22], or enzymes are dissolved and then used for extraction of raw materials [23-25]. Alcalase and neutrase are commercial enzymes and widely used in hydrolysis or protein modification. Alcalase and neutrase have different characteristics of catalytic activity. Alcalase is an active protease, stable at high temperatures, and optimum at alkaline pH conditions. Alcalase can be used immediately after pretreatment to remove non-collagen protein, fat, and minerals from the material without adjusting the pH [19]. Neutrase has been preferred to catalyze hydrophobic amino acids and has many catalyzing sites to break the proteins into peptides [26]. The degradation of collagen by neutrase reportedly starts from the exterior. The enzyme binds tightly to a triple helix at the surface, and molecules in the interior become accessible to the enzyme only in the course of the progressive degradation from the outside. After the triple helix is cracked, its primary fragments are cleaved into small peptides and amino acids [27].

Based on the explanation, there was interest for a further investigation on the potency of enzymatic extraction using alcalase and neutrase for producing gelatin from the *Kacan*g goat bone, identifying the physicochemical characteristics and functional properties of the produced gelatin.

## Materials and Methods

### Ethical approval

No animals were experimented in the present study. Thus, there was no requirement for ethical approval.

### Study period and location

This study was performed from March 2020 to February 2021. Male *Kacang* goat bones were collected from the local slaughtered house of Wonogiri District, Central Java Province. The raw bone materials were stored at the –20°C prior used for the experiment, the skin preparation and gelatin extraction were performed in the Laboratory of Technology of Leather, Animal by-product and Animal Waste, Faculty of Animal Science, Universitas Gadjah Mada. Variable analyses were measured in the same laboratory, excluding the high-performance liquid chromatography (HPLC) for amino acid composition and fourier transform infrared (FTIR), which were performed in Integrated Laboratory for Testing and Research, Univeristas Gadjah Mada.

### Source and sample preparation

*Kacang* goat bones were obtained from a male goat aged 6-12 months. The bone has been separated from the meat and skin in a local slaughterhouse in Wonogiri districts, Central Java Province, Indonesia. The bones were cleaned, placed in polyethylene bags, and stored at −20°C until use. In this study, the protease commercial enzyme was prepared with alcalase and neutrase. The protease enzyme preparations used in this study are shown in Table-1. Bones were thawed in a refrigerator at 4°C for 12 h and cleaned of surface contaminants, fat, meat, and cuticles by soaking the samples in water at 40°C for 30 min; then, any excess remaining flesh was scraped off with a knife. Bone was cut into 5-10 cm per piece, and the marrow was removed. Subsequently, it was dried in the oven at 40°C, and the bone was ground into crumbles shaped at 1-3-mm-sized particles using an iron mortar, weighed, and stored for the next analysis.

**Table 1:** Enzyme products and the conditions of pH and temperature used for preparing goat bone gelatin in this study.

Enzymes	pH, temperature	Source of enzyme	EC Number	Activity
Alcalase 2.4L	pH 8, 50°C	*Bacillus licheniformis*	EC 3.4.21.62	≥2.4 U/g
Neutrase 0.8L	pH 8, 45°C	*Bacillus amyloliquefaciens*	EC 3.4.24.28	≥0.8 U/g

Source: Sigma-Aldrich Chemical Co. (St. Louis, MO, USA)

### Procedures of extraction

#### Pre-treatment

The bone crumbles were prepared and soaked in 0.1 M NaOH with a ratio of 1:5 (w/v) for 48 h to remove non-collagen proteins, and the NaOH solution was changed every 12 h. Thereafter, bone crumbles were cleaned thoroughly with distilled water and then soaked in 10 volumes of 10% (v/v) butyl alcohol for 72 h to remove the fat compound. The butyl alcohol solution was replaced every 12 h. After washing with distilled water, the clean bone crumbles were decalcified with 10 volumes of 0.5 M EDTA-2Na solution (pH=7.5) for 5 days, and the solvent was replaced every 12 h. On completion, the bone crumble sample was washed carefully until clean [28].

#### Gelatin extraction

The bone crumbles were extracted using alcalase and neutrase with four levels of concentration at 0 U/g (AG-0 and NG-0), 0.02 U/g (AG-1 and NG-1), 0.04 U/g (AG-2 and NG-2), and 0.06 U/g (AG-3 and NG-3). The enzymes character and reaction conditions are described in Table-1. The ratio of bone crumbles to distilled water was 1:5 (w/v), and the mixture solution was set following the optimal conditions of each enzyme. The optimal condition of alcalase occurred at pH of 8 and temperature of 50°C for 2.5 h and that of neutrase occurred at pH of 8 and temperature of 45°C for 15 h [5,29]. Furthermore, the mixture was adjusted to pH 7 (neutral); then, enzyme activity was inactivated by heating at 95°C for 15 min; then, the enzyme was extracted for 3 h at 60°C in the water bath. The mixture was filtered using two layers of cheese cloth and continued using a Whatman No. 4 filter paper. The filtered solution was poured in thin layers into plastic trays and dried using a Memmert IN 55 oven at 50°C for 48 h. The resulting dried form of gelatin was collected and then crushed and weighed.

### Physicochemical properties of gelatin

#### Proximate analyses

The proximate analysis, including the moisture, ash, fat, and protein contents, was performed on bone, and gelatin derived from the extracted bone was measured in the proximate analysis according to the procedures of the Association of Official Analytical Chemists [30]. The moisture, ash, and fat contents of the extracted dried gelatin were determined according to the Association of Official Analytical Chemist methods number 927.05, 942.05, and 920.39, respectively. The crude protein content was determined by estimating its total nitrogen content using the Kjeldahl method according to method number 984.13. The protein content was calculated using a conversion factor of 6.25 for the bone protein and 5.55 for gelatin.

#### Gelatin yield

The weight of the wet bone was considered while determining the gelatin yield [31].


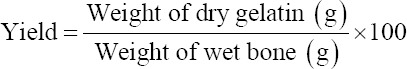


#### pH of gelatin

The pH condition of extracted gelatin was determined according to See *et al*. [32]. A total of 0.667 g of dry gelatin was dissolved in 10 mL of distilled water at 60°C for 30 min using a magnetic stirrer IKA C-MAG HS 7. The gelatin solution was cooled at 25°C (room temperature) for 30 min, and a glass electrode was connected to a pH meter HI 2210 standardized with pH 4.0, 7.0, and 10.0 of standard buffers.

#### MW profile

Gelatin MW was determined using sodium dodecyl sulfate-polyacrylamide gel electrophoresis (SDS-PAGE Atta Pager un AE-6500) according to the method of Laemmli [33]. Gelatin (5 mg/mL) was incubated at 95°C for 5 min and centrifuged at 6000 g× for 1 min. Gelatin samples (10 mL) were mixed with 0.5 HCl Tris buffer (pH 6.8), 5% 2-mercaptoethanol, 20% glycerol, 0.1% bromophenol blue, and 10% SDS gradient. The gel was run at 90 V and then stained with Coomassie Brilliant Blue in 10% acetic acid, 40% methanol, and 40% ddH_2_O.

#### Amino acid profile of gelatin

A sample of 60 mg was added with 4 mL of 6 N HCl, heated for 24 h at a temperature of 110°C, cooled at room temperature, and then neutralized (pH 7) with 6 N NaOH. Then, the sample was added with distilled water to a volume of 10 mL and filtered with 0.2 mm Whatman filter paper. Moreover, 50 mL of the sample plus 300 Orthl of orthophalaldehyde (OPA) solution was stirred 5 min and placed in the Thermo Ultimate 3000 RS HPLC injector and fluorescence detector with 10 mL LiChrospher column and 100 RP-18 column.

#### FTIR spectroscopy

FTIR analysis of dry gelatin from *Kacang* goat bones was performed using Perkin Spectrum One (Shimadzu Prestige 21) according to the method of Renuka *et al*. [34]. The sample and KBr were mixed at a ratio of 1:100 for the preparation of the pellets to measure the absorbance spectrum from 650 to 4000 cm^−1^. The spectral signal was collected in 36 scans at a resolution of 16 cm^−1^. Spectral data analysis was performed using OMNIC 9.0 software (Thermo Fisher Scientific, USA).

### Potency of functional properties from gelatin

#### Melting point

The melting point was conducted according to Kuang and Mohtar [35] with some modifications. Gelatin of 6.67% was prepared in a glass vial by dissolving 0.334 g of dry gelatin in 5 mL of distilled water. This mixture was dissolved at room temperature for 30 min and then heated in a water bath at 60°C for another 30 min. The dissolved samples were cooled at room temperature for 30 min and then chilled in a refrigerator at 7°C for 18 h. The samples were transferred into a water bath at 10°C and inverted. The water bath was warmed gradually by adding warm water at about 1°C per min. The temperature was recorded at which the gel melted.

#### Gel strength measurement

The gelatin sample was dissolved in distilled water at 60°C for 30 min to obtain a final concentration of 6.67% (w/v), and the solution was cooled in a refrigerator at 7°C (maturation temperature) for 16-18 h. The gel strength of gelatin gel was determined at 7°C using a Model TA-TX2 texture analyzer with a 5 kN load cell equipped with a 12.7-mm diameter flat-faced cylindrical Teflon plunger (plunger surface area = 126,728 mm^2^). Gel strength was expressed as maximum force (in g bloom) required for the plunger to press the gel by 4-mm depression at a rate of 1 mm/s. The measurement was performed in triplicate [36].

#### Gelatin viscosity

Gelatin solutions at the concentration of 6.67% (w/v) were prepared by dissolving the dry powder in distilled water and heating at 60°C for the determination of viscosity. Moreover, the viscosity (cP) of the 10 mL solution was determined using a texture analyzer CT3 viscometer (Brookfield) equipped with a spindle No. 61 at 60 rpm [37].

#### Foaming properties

Foam expansion (FE) and foam stability (FS) of gelatin extracted by alcalase (AG) and neutrase (NG) were determined following Ktari *et al*.’s [31] method with slight modifications. Moreover, 50 mL of gelatin solution (1% w/v) was transferred into 250-mL cylinders and homogenized with an Armfield L4R at a speed of 13,400 rpm for 1 min at room temperature. The sample was allowed to stand for 0 and 30 min.

FE was expressed as a percentage of volume increase after homogenization at 0 min, which was calculated according to the following equation:


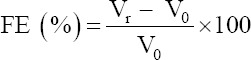


FS was calculated as the volume of foam remaining after 30 min.


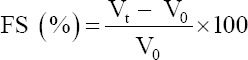


where, Vr = Total volume after whipping; V0 = The original volume before whipping, and Vt = Total volume after leaving at room temperature for 30 min.

#### Water holding capacity (WHC) and oil holding capacity (OHC)

WHC and OHC were measured using the following method by Cho *et al*. [38] with slight modifications. One gram of gelatin was placed in a centrifuge tube and weighed (tube with gelatin). For measuring WHC and OHC, 50 mL distilled water or 10 mL soybean oil were added, respectively, and held at room temperature for 1 h. The gelatin solutions were mixed with a vortex mixer for 5 s every 15 min. The gelatin solutions were centrifuged at 450 g for 20 min. The upper phase was removed, and the centrifuge tube was drained for 30 min on a filter paper after tilting to a 458° angle. Their capacities were calculated as the weight of the contents of the tube after draining divided by the weight of the dried gelatin and expressed as the weight percent of dried gelatin.

### Statistical analysis

The data of proximate, yield, pH, and functional properties from gelatin were analyzed using one-way analysis of variance, and the mean differences were tested using Duncan’s multiple range test. The statistical analysis was performed using SPSS version 26. Results showed significance at p<0.05. MW, amino acid profile, and FTIR spectroscopy of gelatin were analyzed descriptively. Data are presented as mean ± standard error of the mean.

## Results

### Proximate analysis

The proximate composition of the goat’s bones includes moisture, ash, fat, and crude protein content of 9.19, 49.43, 10.10, and 22.12%, respectively. Furthermore, the composition of *Kacang* goat bone gelatin extracted using alcalase (AG) and neutrase (NG) enzymes at different levels is shown in Table-2. Alcalase and neutrase enzymes affect (p<0.05) the moisture content of gelatin. The moisture content of alcalase treatment varied greatly between treatments and was highest in samples GA-1 and GA-2, followed by GA-3 and GA-0 (p<0.05). Alcalase and neutrase enzymes reduced gelatin ash content. The lowest fat content (p<0.05) was indicated by AG-1, followed by AG-2 and AG-3. The protein content of gelatin samples extracted with alcalase enzyme increased compared to no alcalase treatment but did not change at all alcalase levels. Extraction with neutrase increased the protein and fat content of gelatin. The addition of neutrase did not affect the fat content but affected the gelatin protein content. The protein content of NG-3 was higher than that of NG-1 and NG-2.

**Table 2:** Proximate composition of the *Kacang* goat bone gelatin extracted using enzymes alcalase (AG) and neutrase (NG) at different levels.

Parameter	Gelatin source	Enzyme Treatment (U/g)			

		0 (Blank)	0.02	0.04	0.06
Moisture (%)	AG	9.39±0.21^b^	9.94±0.08^a^	9.92±0.08^a^	9.47±0.06^b^
	NG^ns^	9.22±0.24	9.15±0.05	9.20±0.13	9.24±0.06
Ash (%)	AG	15.63±0.69^a^	1.66±0.01^b^	1.90±0.02^b^	1.61±0.03^b^
	NG	17.29±0.05^a^	2.81±0.33^b^	2.93±0.08^b^	3.00±0.08^b^
Fat (%)	AG	0.76±0.01^a^	0.50±0.02^c^	0.68±0.02^b^	0.69±0.01^b^
	NG^ns^	1.43±0.05	1.45±0.10	1.38 ± 0.08	1.53±0.15
Protein (%)	AG	31.23±1.04^b^	70.21±0.03^a^	69.96±0.25^a^	69.65±0.28^a^
	NG	32.30±0.02^c^	69.93±0.05^b^	69.52±0.52^b^	70.89±0.28^a^

AG=Alcalase gelatin, NG=Neutrase gelatin. ^a,b,c,^The different alphabet superscripts in the same row indicate significant difference (p<0.05). ^ns^=Non significant

### Yields and pH

The percentage of yield and pH of *Kacang* goat bone gelatin extracted using alcalase (AG) and neutrase (NG) at different levels are shown in Table-3. There was a significant increase in the yield of gelatin extracted with alcalase and neutrase enzymes. The addition of enzymes during the extraction process increases the yield by more than 100%. The yield of AG-3 (9.78±0.86%) was significantly (p<0.05) higher at all levels of alcalase treatment and with neutrase at NG-3 (6.35±0.09%). There was a significant decrease in pH in gelatin with alcalase and neutrase. The pH of gelatin without alcalase (7.10) and neutrase (7.15) were highest (p<0.05) between treatments.

**Table 3:** The yield and pH value of the *Kacang* goat bone gelatin extracted using enzymes alcalase (AG) and neutrase (NG) at different levels.

Parameter	Gelatin source	Enzyme Treatments (U/g)			

		0 (Blank)	0.02	0.04	0.06
Yields (%)	AG	0.88±0.08^c^	5.67±0.37^b^	9.68±0.56^a^	9.78±0.86^a^
	NG	0.71±0.04^d^	3.18±0.28^c^	5.33±0.13^b^	6.35±0.09^a^
pH value(%)	AG	7.10±0.06^a^	6.21±0.04^b^	6.25±0.07^b^	6.29±0.03^b^
	NG	7.15±0.03^a^	6.19±0.17^b^	6.28±0.06^b^	6.32±0.02^b^

AG=Alcalase gelatin, NG=Neutrase gelatin. ^a,b,c,d^The different alphabet superscripts in the same row indicate significant difference (p<0.05). ^ns^=Non significant

### MW profile

**Figure-1:**
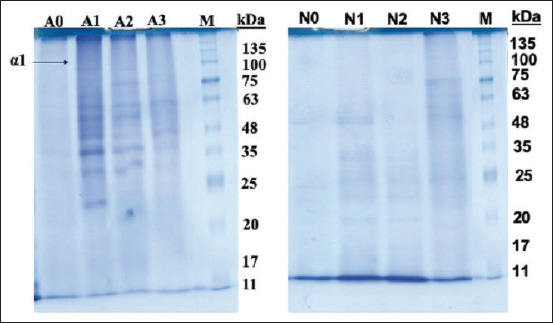
Sodium dodecyl sulfate-Page paten of gelatin extracted from the *Kacang* goat bone using alcalase (a) and neutrase (b) enzymes. Lane M: Marker; Lane A0 and N0: gelatin with alcalase and neutrase blank; Lane A1 and N1: gelatin with alcalase and neutrase 0.02 U; Lane A2 and N2: gelatin with alcalase and neutrase 0.04 U; Lane A3 and N3: gelatin with alcalase and neutrase 0.06 U.

Polyacrylamide gel electrophoresis with SDS was used to analyze the protein profile of gelatin. The estimated MW distribution of *Kacang* goat bone gelatin with alcalase and neutrase at different concentrations was compared and presented in Figure-1. In gelatin with alcalase or neutrase, there was no band indicating collagen components, such as β and γ-chains. Alcalase enzyme treatment showed that gelatin contains high-intensity α-chain polypeptides mainly in AG-1 and AG-2 with MW of approximately 105 kDa, and other fractions with lower MWs are more clearly formed. Gelatin protein bands with neutrase formed high-intensity fractions noted in NG-3 with a MW of approximately 75 kDa and several other low MW fractions. There was no visible protein band in gelatin without alcalase (AG-0) or neutrase (NG-0) enzyme treatment.

### Profile of amino acid composition

The amino acid composition of *Kacang* goat bone gelatin after enzymatic extraction with alcalase and neutrase at different concentrations is presented in Table-4. The amino acid content of *Kacang* goat bone gelatin extracted with alcalase and neutrase has a similar composition. Glycine as the dominant amino acid accounts for approximately one-third of the amino acid profile, followed by alanine, aspartic acid, and glutamic acid. Gelatin extracted with alcalase and neutrase showed low tyrosine and methionine content and did not contain tryptophan, histidine, and cysteine. Gelatin without alcalase (AG-0) and neutrase (NG-0) enzymes showed higher aspartic acid and glutamic acid content than gelatin with enzyme treatment.

**Table 4:** Amino acid composition (%) of gelatin extracted using alcalase (AG) and neutrase (NG) enzymes from the *Kacang* goat bone.

Amino acid	Levels of enzyme alcalase (AG) (U/g)				Levels of enzyme neutrase (NG) (U/g)			
	
	0	0.02	0.04	0.06	0	0.02	0.04	0.06
Aspartic acid	9.01	5.11	4.93	5.25	8.13	7.20	6.84	6.81
Glutamic acid	8.83	6.66	6.54	6.15	7.26	8.26	6.63	6.55
Serine	1.47	2.97	2.95	1.89	1.24	0.69	1.35	1.28
Glycine	27.50	29.30	29.14	28.18	28.00	29.10	29.24	28.48
Threonine	0.88	0.82	1.98	1.99	1.38	1.14	1.01	1.29
Arginine	3.39	2.00	3.11	3.28	3.52	2.11	2.27	2.31
Alanine	8.00	8.53	8.42	8.51	8.11	8.13	8.61	8.12
Tyrosine	0.17	0.89	0.76	0.82	0.62	0.52	0.65	0.71
Methionine	0.21	0.61	0.34	0.31	0.31	0.91	0.31	0.28
Valine	2.00	2.81	3.05	3.01	2.42	2.21	2.86	3.01
Phenylalanin	0.72	1.72	1.67	1.00	0.61	0.98	1.41	1.55
Isoleucine	0.93	1.62	1.52	2.52	1.33	1.29	1.68	1.64
Leucine	2.03	2.12	2.19	2.02	1.67	2.05	2.07	2.36
Lysin	0.85	1.56	1.60	0.95	0.75	1.86	1.73	1.67

Values are means (n=2), for descriptive analysis

### FTIR spectra

**Figure-2:**
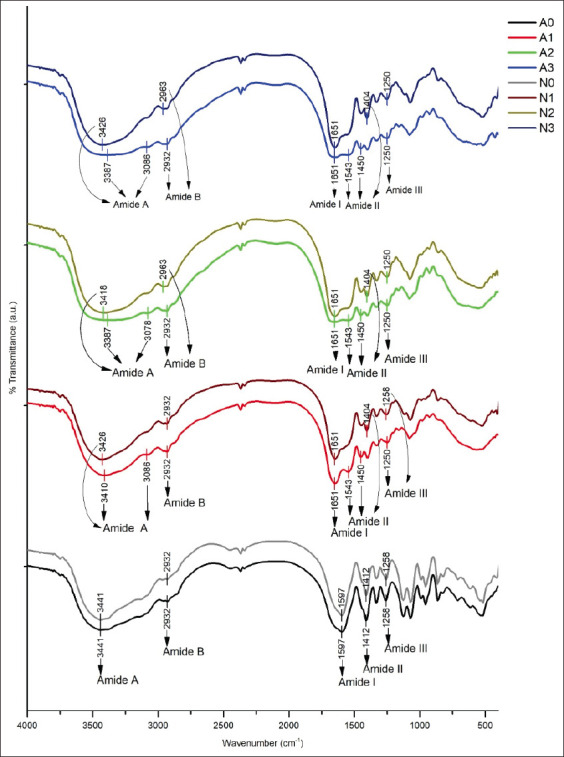
Fourier transform infrared spectrum of *Kacang* goat bones extracted with alcalase and neutrase. A0 and N0: gelatin with alcalase and neutrase blank; A1 and N1: gelatin with alcalase and neutrase 0.02 U; A2 and N2: gelatin with alcalase and neutrase 0.04 U; A3 and N3: gelatin with alcalase and neutrase 0.06 U.

The FTIR spectra of gelatin from *Kacang* goat bone extracted with alcalase and neutrase at different concentrations are presented in Figure-2. Enzyme-extracted gelatin derived from *Kacang* goat bones represents the same spectrum, but the spectrum for enzyme-free gelatin is different. FTIR spectroscopy is a non-destructive and rapid technique, which has been used to elucidate changes in the secondary structure of proteins during the transformation of collagen to gelatin, compared to FTIR spectra of gelatin (Table-5). Amide A band of *Kacang* goat bone gelatin with alcalase appeared at all levels of enzyme concentration, recorded at 3441, 3410, 3387, and 3387 cm^−1^ for AG-0, AG-1, AG-2, and AG-3, respectively, which were close to the free N-H stretching frequency. Gelatin with neutrase has a lower frequency wavenumber than gelatin with alcalase, namely, 3426, 3418, and 3426 cm^−1^ for NG-1, NG-2, and NG-3, respectively. Amide band B in the samples AG-0, AG-1, AG-2, and AG-3 was recorded at the wavenumber with the same frequency, namely, 2932 cm^−1^. Gelatin amide I band with alcalase and neutrase showed the same frequency wavenumber, namely, 1651 cm^−1^, but it appears that AG-1 and NG-3 have sharper and more prominent absorption bands. This range of frequencies is known as the α-helix. AG-0 and NG-0 do not have C = O absorption. In the amide II area, the gelatin absorption band characteristics of all alcalase concentrations were noted at 1543 cm^−1^. The amide II wavenumber of gelatin with neutrase ranged far below that of gelatin with alcalase, which is 1404 cm^−1^. Furthermore, both AG-0 and NG-0 have the same wavenumber, which is 1597 cm^−1^. The amide III bands were observed in gelatin with alcalase and neutrase with the same wavenumber (1250 cm^−1^), appearing in gelatin with all levels of alcalase (AG-1, AG-2, and AG-3) and then with neutrase at NG-2 and NG-3, lower than AG-0, NG-0, and NG-1 (1258 cm^−1^).

**Table 5:** Fourier transform infrared peak spectra of *Kacang* goat bone gelatin extraction by alcalase (AG) and neutrase (NG).

Region	Peak wavenumber (cm^-1^)							

	Levels of enzyme alcalase (AG) (U/g)			Levels of enzyme neutrase (NG) (U/g)				
	
	0	0.02	0.04	0.06	0	0.02	0.04	0.06
Amide A	3441	3410	3387	3387	3441	3426	3418	3426
Amide B	2932	2932	2932	2932	2932	2932	2939	2939
Amide I		1651	1651	1651		1651	1651	1651
Amide II	1597	1543	1543	1543	1597			
		1450	1450	1450	1412	1404	1404	1404
Amide III	1258	1250	1250	1250	1258	1258	1250	1250

### Functional properties of *Kacang* goat bone gelatin

Functional properties of *Kacang* goat bone gelatin extracted with alcalase and neutrase at different concentrations included melting point, gel strength, viscosity, FE, FS, WHC, and OHC, which are presented in Table-6. Alcalase and neutrase treatments increased the functional properties of gelatin, except that the viscosity remained at all alcalase levels. The highest melting point, gel strength, and WHC of gelatin were shown by AG-1 and AG-2 (p<0.05), but the highest FE and FS of gelatin were observed in AG-1 and AG-2 with the highest OHC. Gelatin with neutrase had the highest melting point, FE, and OHC (gelatin in NG-2), but gel strength, viscosity, FE, and WHC did not differ at all alcalase levels. The addition of alcalase and neutrase did not increase the OHC of gelatin.

**Table 6:** Functional properties of *Kacang* goat bone gelatin extracted using alcalase (AG) and neutrase (NG).

Parameter	Gelatin source	Enzyme treatments (U/g)			

		0 (Blank)	0.02	0.04	0.06
Melting point (^o^C)	AG	26.40±0.43^c^	28.47±0.40^a^	28.33±0.25^a^	27.47±0.15^b^
	NG	25.50±0.46^c^	27.47±0.25^ab^	27.63±0.11^a^	26.83±0.47^b^
Gel strength (g bloom)	AG	55.18±0.32^c^	68.14±0.55^a^	67.41±0.07^a^	66.92±0.64^b^
	NG	52.94±0.26^b^	57.42±0.46^a^	56.83±0.34^a^	57.27±1.03^a^
Viscosity (Cp)	AG^ns^	2.33±0.15	2.80±0.17	2.40±0.56	2.37±0.25
	NG	1.82±0.07^b^	2.03±0.25^a^	2.00±0.36^a^	2.21±0.31^a^
FE (%)	AG	17.50±1.25^d^	71.67±1.84^a^	64.33±2.31^b^	43.33±2.31^c^
	NG	11.33±2.31^c^	69.33±10.60^a^	83.33±3.51^a^	78.33±1.15^a^
FS (%)	AG	9.17±2.89^c^	52.67±2.52^a^	30.00±2.94^b^	12.33±4.93^c^
	NG	4.67±1.15^c^	46.33±4.04^b^	61.67±5.13^a^	47.33±5.03^b^
WHC (%)	AG	177.33±7.02^c^	334.67±3.05^a^	324.00±6.00^a^	269.33±6.43^b^
	NG	239.00±3.84^b^	324.11±3.84^a^	302.78±3.99^a^	284.00±2.00^a^
OHC (%)	AG	152.00±7.21^d^	242.33±8.62^b^	283.33±5.77^a^	171.00±1.00^c^
	NG	165.00±1.00^c^	216.67±7.37^b^	265.33±4.51^a^	152.33±6.80^c^

AG=Alcalase gelatin, NG=Neutrase gelatin. ^a,b,c,d^The different alphabet superscripts in the same row indicate significant difference (p<0.05). ^ns^=Non significant

## Discussion

Gelatin is a denaturalized protein derived from collagen and can be extracted from the bone or skin. In this study, *Kacang* goat bones were used as raw materials. The results of the proximate analysis of goat bones showed that the ash content was 50% higher than that of other bone constituents, namely, 49.43%. Moreno-Piraján *et al*. [39] reported that bone consists of 60% inorganic components (hydroxyapatite), 10% water, and 30% organic components (bone matrix proteins). *Kacang* goat bone extraction using alcalase and neutrase increased the gelatin yield compared to those without enzymes. The increase in gelatin yield is related to the amount of collagen that can be converted into gelatin. Enzymes can decompose collagen fibers more quickly and convert triple-helical collagen fibers into single chains [40]. The highest yield was observed on AG-2 (9.68±0.56%) and AG-3 (9.78±0.86%), followed by NG-3 (6.35±0.09%). The increase in gelatin yield with increasing alcalase and neutrase concentrations was related to the amount of collagen that could be converted into gelatin. Collagen substrate is still available, so that the process of cleavage of bone collagen peptides is still ongoing. Enzymes can break down collagen fibers more quickly and convert triple-helical collagen fibers into single chains, and when the amount of enzyme increases, the hydrolysis speed is faster and tends to increase the conversion of collagen to gelatin [41]. The yield of gelatin extracted from Peking duck legs was higher (17.94% based on dry weight) compared to acid and base [21], and Cao *et al*. [42] reported that the gelatin yield of bovine bone collagen incubated with pepsin (6.73-11.75%) was higher than that without pepsin. The higher gelatin yield reported from goat skin was 15.57-23.28% [43]. The skin has higher collagen than bone with a more dominant ash content [44]. The higher yield of gelatin extracted with alcalase than neutrase indicated the ability of alcalase to hydrolyze a wider range of protein substrates. Alcalase produces many hydrolytic enzymes to degrade many different substrates and has broad specificity for cleavage of peptide bonds and shrinking of the triple helix structure of collagen in a short time [40]. Moreover, alcalase is a multifunctional enzyme (can also act as an esterase and can potentiate efficient stereoselective hydrolysis of amino esters, as well as transesterification and transpeptidation reactions).

Determining the pH of gelatin is important because it can limit the use of gelatin in the product. The high pH of AG-0 and NG-0 was caused by the accumulation of chemicals in the demineralization process using EDTA-Na pH 7.5, which was not completely washed off during the neutralization process. Acid and base treatment is required to separate proteins, fats, and non-collagen minerals, before water extraction, and depending on the type of acid or base and the concentration used, this pre-treatment greatly affects the final pH of gelatin [14]. The treatment of alcalase and neutrase enzymes decreased the pH of gelatin at all enzyme concentrations. This is presumably because the enzyme helps the extraction process. The pH will decrease when extraction occurs at pH >6.5, due to the decomposition of the amino groups released from the cleavage of the peptide bonds [45]. In contrast to gelatin without enzyme treatment, the peptide bond has not been completely broken, so the initial pH of the extraction and pretreated inorganic substances are carried over to the final result. Different pH values were reported for gelatin from various other sources: Gelatin from Peking duck feet treated with alcalase yielded a pH of 6.94 [21], that from camel bone with acid demineralization 5.3 [13], and that from chicken feed bone 6.15 [46].

The moisture content in food is one of the important chemical components because it determines the texture and shelf life, especially against microbial damage. The moisture content of goat bone gelatin ranges from 9.15% to 9.94%, <15% prescribed for edible gelatin [35], while water content >16% is undesirable because of the risk of microbial growth [8]. Variations in the moisture content of gelatin samples can be caused by inadequate drying and storage processes. Conditions during the drying process, such as unstable temperature, sample density in the oven, humidity during storage, and moisture permeability in storage containers, are the causes of high or low gelatin water content [44].

The high ash content in the sample without enzyme treatment was thought to be due to the high ash content of *Kacang* goat bone (49.43%), which is used as raw material for gelatin, which is difficult to remove. Bone collagen biomass is a 3D space from the mineral phase (nanoparticles from calcium phosphate minerals) that was scattered in all collagen structural arrangements in bone [47]. Another cause of high ash content was the presence of inorganic salts produced during the pretreatment [36]. Enzyme treatment was able to reduce gelatin ash content. This is assumed because proteolytic enzymes have catalytic specificity and are able to degrade collagen in bone and separate other proteins and inorganic compounds. Ma *et al*. [17] used pepsin to demineralize pork bones and showed high yield, which is associated with perfect pretreatment. The ash content of the Aceh cattle scapular bone gelatin reported by Jelita *et al*. [14] with decalcification for 8 days and extracted from the acid was 4.67%. Abedinia *et al*. [21] reported that ash content of Peking duck leg gelatin with alcalase was 2.1% and those of gelatin pretreatment from metatarsus, claws, tendons, and chicken claws were 6.03, 12.28, 6.38, and 1.92%, respectively [8].

As with ash, bone fat was also removed during the pretreatment process of bone before gelatin extraction. The fat removal process before extraction affects the characteristics and functional properties of the resulting gelatin [48]. Soaking the bone raw material with hot water to facilitate the removal of meat scraps and other contaminants during the bone raw material preparation process, followed by the fat removal process with butyl alcohol and extraction using distilled water at hot temperatures, causing the fat to collect on the surface of the gelatin solution and retain in filter paper during the filtering process, decreasing the levels of gelatin fat. The low proportion of fat content, followed by numerically high protein content, especially in AG-1 indicates that the removal of bone fat has been conducted efficiently. Protein solubility during hydrolysis, removal of undigested non-protein substances, and removal of some fat after hydrolysis result in high protein content in hydrolysis [49,50]. The fat content of freshwater fish gelatin extracted by alcalase and pretreatment using isobutyl alcohol was 7.93 g/100 g gelatin [11], and the gelatin protein content of chicken feet was 67.40% [46].

The main factor affecting the gelatin protein pattern is hydrolysis during the extraction process, which contributes to the separation of the peptide chains [51]. The absence of b and g chains of gelatin in this study indicated that most cross-links between the chains were broken during the extraction process. There has been extensive hydrolysis of collagen during the gelatin production process, particularly hydrolysis with neutrase. Neutrase specifically breaks down collagen molecules to form hydrolyzates [27]. These results are consistent with the study by Lassoued *et al*. [52], who used fish skin gelatin to produce protein hydrolyzate and found that hydrolyzate had the best ACE and DPPH radical inhibitory activity with neutrase from *Bacillus amyloliquefaciens* compared to A2 from *Bacillus subtilis*. The indistinct results of protein MW measurements using SDS-PAGE from AG and NG samples may be caused by the lack of control at extraction time, related to pH, enzyme concentration, or extraction time. Zhang *et al*. [40] found a-chains with a MW of 116 kDa of spiked skate skin gelatin (*Amblyraja radiata*) by alcalase pretreatment and controlled extraction. Furthermore, it is stated that the a chains are easily degraded in liquid solvents, so it is extremely important to strictly control the processing conditions to obtain the intact a-chains to suit a particular application [53].

The composition of the gelatin amino acid profile by HPLC using OPA solution for precolumn derivatization resulted in 15 primary amino acids. Rafieian *et al*. [54] reported that glycine is the dominant amino acid in the residual gelatin to remove chicken manure (311.5 mg/g), which has low levels of isoleucine, methionine, and tyrosine, without tryptophan, histidine, and cysteine. High aspartic acid and glutamic acid can be caused by a series of bone pretreatment processes with EDTA pH 7.5, followed by imperfect neutralization and enzymatic extraction at optimum pH, especially alkaline. According to Zhou and Regenstein [55], the conversion of collagen to gelatin causes changes in some amino acid molecules. Alkali processes can deaminate glutamine to glutamic acid and asparagine to aspartic acid so that the composition of aspartic acid and glutamic acid is higher, which causes a decrease in the number of amide groups and an increase in carboxyl groups in gelatin molecules [56].

Spectroscopy is usually used to see functional groups and secondary structures of gelatin proteins. The exact location depends on the hydrogen bonds and configuration of the protein structure [57]. Amide A represents the N-H strain associated with hydrogen bonds, which can be observed in the wave range 3400-3440 cm^−1^ [58]. When the enzyme concentration increased at AG-2 and AG-3, as well as all levels of neutrase concentration, the amide A band shifted to a lower frequency. These results are similar to those of Abedinia *et al*. [21] on enzymatically extracted Peking duck gelatin. This indicates a further change in collagen structure, leading to gelatin degradation. The lower frequency wavenumbers indicate changes in the secondary collagen structure and hydrogen bonds between the N-H groups of the shorter peptide fragments [59]. The hydrogen bond interaction causes a decrease in the wavenumber in amide A [60]. The lower frequency band of amide A in gelatin with neutrase indicates that there has been extensive degradation of collagen by neutrase. Neutrase specifically breaks down collagen molecules to form hydrolysates [27]. The stretching of the C = O carbonyl double bond, with the contribution of the bending in the N–H bond phase and the C–N bond strain, occurs in the frequency range of the 1660-1620 cm^−1^ region, which is often referred to as the amide 1 band. The frequency range 1660-1650 cm^−1^ is known as the a-helix [61]. This is clear when associated with protein bands, the SDS-PAGE results, showing that gelatin with alcalase and neutrase contains only a-chains, whereas b-chains have been degraded by enzymes. The amide II wavenumber formed by gelatin with an alcalase (1543 cm^−1^) has an a-helical structure and a lower wavenumber neutrase formed as a result of peptide formation. The range of wavenumbers 1550-1520 cm^−1^ is due to amide II, with an a-helical structure in the range of 1550-1540 cm^−1^. The amide II vibrations were associated with a model outside of the combined C-N strain coupled with the NH deformation of the peptide group [56]. The low amplitude in the amide III band indicates a greater disturbance in the molecular structure due to the transformation of the a-helix into the coil structure due to the addition of the alcalase enzyme and increase in the neutrase concentration. Muyonga *et al*. [58] stated that this change was associated with the loss of the triple helix state as a result of the denaturation of collagen to gelatin.

The functional properties of gelatin are related to gel formation and surface formation of gelatin molecules [62]. The melting point is another important property of gelatin, and the specific temperature degree when the gel becomes soft or melts is recorded as the melting point. There was an increase in the melting point, presumably the proportion of amino acids and protein MW in AG-1 and AG-2, which formed and stabilized the gelatin structure. Furthermore, it is directly related to the viscosity and strength of the gelatin gel, which are high. Rahman and Jamalulail [46] found that chicken leg gelatin with low gel strength and viscosity resulted in a low melting point temperature. Different results for gelatin melting point from sheep skin with alcalase extraction were measured at a temperature of 15-27°C [25]. Gel strength is a function of the complex interactions determined by the amino acid composition and ratio of the a and ß chain components. The strength of AG-1 and AG-2 was higher compared to that of gelatin without alcalase. This is thought to be related to the crude protein content of gelatin (69.65-70.21%), which contains the amino acids that form the structure of gelatin. The strength of the gel depends on the protein concentration. Proteins consist of amino acids, which are responsible for the formation of a stable triple helix structure that directly involves the formation of hydrogen bonds between free water molecules and amino acid hydroxyl groups in gelatin [63,64]. There was a decrease in the strength of the AG-3 gel, presumably due to collagen bonds, which may have further degradation with the addition of alcalase resulting in protein denaturation. The complete denaturation process of the collagen molecules can lead to a decrease in gel strength [65]. Gelatin with neutrase showed that, at all concentrations of added neutrase, it was unable to increase gel strength or that the gelation process with neutrase was very low. Damrongsakkul *et al*. [27] used a neutrase to extract the gelatin from raw skin and found that the protein could not form gelatin, which is presumably because the neutrase specifically breaks down collagen molecules to form a hydrolyzate. This is confirmed by the study of Hosseini-Parvar *et al*. [66] stated that the interaction of concentration and treatment time with neutrase did not affect the strength of the gelatin in the bovine bone. Akagündüz *et al*. [11] reported that the strength of freshwater fish bone gelatin gel with HCl treatment (87.3 g bloom) was higher than that of alcalase (81.7 g bloom).

Viscosity is also an important characteristic of commercial gelatin and varies widely, depending on factors, such as MW, gel strength, pH, and temperature [67]. The viscosity of *Kacang* goat bone gelatin with enzyme treatment still meets the standards of 1.5-7.5 cP and 2 cP for edible type B gelatin [68]. The lower viscosity of gelatin is thought to be due to the presence of low MW peptide chains as a result of excessive collagen hydrolysis during the extraction stage. Low viscosity gelatin solutions typically produce short, brittle gels, while high-viscosity gelatin solutions produce tough, stretchable gels of high commercial value [53]. Viscosity is also related to pH. Gelatins with high pH usually have low viscosity. In general, the low viscosity of gelatin is found in the pH range 6.5–8 [8]. The different viscosity values of gelatin from the study reported by Norziah *et al*. [69], the by-product of the surimi process with the enzyme bromelain was 1.9 mPa.s.

Foaming properties are important for various food and beverage applications as measured by FE and FS. In general, foam formation is influenced by displacement, penetration, and changes in the composition of protein molecules (source, intrinsic properties, protein composition, and conformation) in solution and on the surface of the air-water phase [70]. The high levels of FE and FS in gelatin were thought to be due to the high content of crude protein, especially in all gelatin extracted with enzymes. At higher protein concentrations, the foam has higher concentration and stable due to the increase in the thickness of the phase surface layer [71]. The decrease in FE and FS in AG-2 and AG-3, possibly due to increasing of enzyme level. This result is in line with those of the study by Jridi *et al*. [72], who reported that the FE from previously treated cuttlefish shell gel with different pepsin concentrations decreased with increasing pepsin concentration. Gelatin with neutrase treatment has a low MW, making it easier to move to the surface phase more quickly. Peptides with smaller MWs can reach the air-water surface phase, resulting in better FE [73]. FE and FS were different from extracted bovine bone gelatin with acetic acid at 150.66% and 114.66%, respectively [74].

WHC and OHC are important functional properties in the food system. This ability is closely related to the texture and taste of the food through interactions between water, oil, and other components. The interaction of water with protein is greatly important in the food system because it is related to the ability of proteins to absorb and retain water against the force of gravity in its components [36]. The study showed that alcalase and neutrase treatment significantly increased the WHC and OHC of gelatin compared to the absence of the enzyme. The same results were obtained with gelatin extracted from fish (*Liza aurata* viscera) with endogenous proteases [75]. Increased WHC and OHC in gelatin with enzymes presence, presumably due to enzyme degraded the bone collagen, causing microstructure and conformational changes in proteins. The increase in WHC in AG-1 and AG-2 was thought to be due to the increase in the proportion of protein and percentage of hydrophilic amino acids. The high WHC gelatin is strongly influenced by the content of hydrophilic amino acids and hydroxyproline, which form gelatin peptides [76]. OHC is more influenced by aromatic amino acids. Alcalase has high specificity for aromatic amino acids, and most of its peptides are hydrophobic. Most hydrophobic amino acids in AG-2 have a higher proportion. The ability of alcalase to break covalent bonds in aromatic and hydrophobic amino acid residues so that it is more open and binds to oil. The levels of exposure of hydrophobic amino acids and tyrosine determine the level of OHC [77]. Low OHC indicates a decrease in hydrophobic interactions due to extensive proteolysis, which causes hydrolytic degradation of protein structures [78]. Neutrase has many catalyst sites for protein breakdown [79]. The amino acid composition of all gelatins with enzyme treatment had similar values, which did not affect the OHC even though alcalase and neutrase were added. The binding of fat and water to proteins is influenced by intrinsic factors, such as size, shape, amino acid composition, protein conformation, and surface hydrophobicity/polarity [80].

## Conclusion

*Kacang* goat bones can be extracted with warm water, which is incubated using alcalase and neutrase enzymes, producing gelatin with different characteristics. Gelatin extraction using alcalase (0.02-0.04 U/g bone) produced gelatin with higher physical, chemical, and functional properties than that of neutrase. The increase in alcalase level to 0.06 U/g has no positive effects of increasing the gelatin quality. Neutrase tends to dissolve the protein at a lower MW and consequently, the gelatin quality tends to decrease. To optimize the gelatin extraction method, it is important to find and explore the other sources of protease enzymes that are cheaper and easily isolated.

## Authors’ Contributions

DM: Contributed to gelatin isolation, enzyme preparation, FTIR analysis, and writing of the manuscript. YE: Designed the experiments, handled the SDS PAGE, analyzed the data, and writing of the manuscript. ES and NN: Contributed to the physical characteristic analysis, analyzed the data, and reviewed draft of the paper. TRH and MZA: Prepared samples, data analysis, writing of the manuscript and contributed in SDS PAGE analysis. All authors have checked and approved the final version of the manuscript.
